# The Effects of Experimental Whole-Body Burning on Histological Age-at-Death Estimation from Human Cortical Bone and Dental Cementum

**DOI:** 10.3390/biology11111569

**Published:** 2022-10-26

**Authors:** Sophia R. Mavroudas, Lauren A. Meckel, Timothy P. Gocha, Justin Z. Goldstein, Shelby L. Garza

**Affiliations:** 1 Department of Anthropology, Forensic Anthropology Center, Texas State University, 601 University Drive, San Marcos, TX 78666, USA; 2 Department of Cell Biology and Anatomy, Health Sciences Center, Louisiana State University, 1901 Perdido Street, New Orleans, LA 70112, USA; 3 New York City Office of Chief Medical Examiner, 520 1st Avenue, New York, NY 10016, USA

**Keywords:** thermal alteration, bone histology, age-at-death, histomorphometry, histotaphonomy

## Abstract

**Simple Summary:**

The objective of this study is to explore the effects of thermal alteration on the microstructure of human bones and teeth through whole-body experiments in various fire-death scenarios. Understanding how thermal alteration can affect microstructure has implications for the use of microscopic analysis in applied contexts including for human vs. nonhuman differentiation, age-at-death estimation, season-of-death estimation, and histotaphonomic interpretation. The results of this study show some microscopic changes post-burning, however, no discernable patterns related to temperature or time of burning were established. The results also showed that histological age-at-death estimation methods could be applied to bones and teeth post-burning. These results also show that the presence and amount of soft tissue on remains affect the degree of burning and the amount of bone remaining for analysis.

**Abstract:**

Whole-body donations (*n* = 6) were placed in various experimental fire-death scenarios to understand the histological effects of thermal alteration on bones and teeth. Midshaft samples of the femur, 6th rib, and metatarsal were removed from each donor pre- and post-burning to examine histomorphometric differences and test established age-at-death estimation methods. Dental samples were taken post-burning to test the applicability of dental cementum analysis for age-at-death estimation. Significant differences in osteon area or Haversian canal area between some pre- and post-burn samples were found although no patterns related to temperature or element were observable. The femoral age estimates across pre- and post-burn samples were 91% accurate across all donors. The point age estimates from the ribs compared to known age were significantly different (t(10) = 6.88, *p* < 0.001) with an average difference of −18.53 years. Dental age estimates of post-burn samples were not significantly different from the known donor age (t(3) = −0.74, *p* = 0.512) with an average difference of −3.96 years. Overall, the results of this study show that thermally altered remains can be used for histologic age-at-death analysis of cortical bone and dental cementum, within certain burning parameters.

## 1. Introduction

The discovery of thermally altered remains in applied contexts can cause complications for forensic investigations and archaeological site interpretation. These complications occur at varying levels and can include the initial processing of the site, the application of anthropological methods, and the final identification. Although research into the analysis of thermally altered human remains is extensive when considering scene or site recovery and gross morphological anthropological analysis [1,2,3,4], there has been less investigation into the applicability of histological methods for thermally altered remains. The aim of this paper is to gain a better understanding, through whole-body experimentation, of histological changes to bones and teeth from thermal alteration in order to accurately utilize histological methods for age-at-death estimation in applied contexts.

### 1.1. The Use of Skeletal Histology in Applied Contexts

Although there are many uses for histological analysis of bones and teeth in applied contexts broadly, microscopic analysis of bone in applied contexts most frequently focuses on analysis of bone type as well as cortical bone structures such as osteons (discrete remodeled units of cortical bone) for human vs. nonhuman differentiation [5,6,7,8] and age-at-death estimation [9,10,11,12,13,14,15,16,17,18,19,20,21,22,23,24,25,26,27,28,29,30,31,32,33]. This analysis often involves the observation of the presence and/or absence of osteons as well as their number and size. Microscopic analysis of teeth in applied context is most frequently used for estimating age [34,35,36,37,38,39] or season-of-death [38,40,41,42] through examination of tooth root characteristics.

#### 1.1.1. Human vs. Nonhuman Differentiation

For highly fragmented or taphonomically altered material, microscopic human/nonhuman differentiation can be assessed through identification of bone type [5,6] or through the use of histomorphometry, which is the quantification of microscopic structures [7,8]. Generally, the practitioner relies on visualization of bone type through 2D microscopic observation either directly through the light microscope or, more often, through digital microscopic images of sufficient quality. The identification of plexiform bone (bone with a layered brick-like appearance) or numerous lines of osteon banding (a row of five or more primary or secondary osteons) can provide a definitive designation of nonhuman for the bone sample in question [5]). Alternatively, measurements of size and circularity of osteons can also be used to distinguish human from nonhuman bone whereby nonhuman osteons are smaller and more circular than human osteons [7]. Thermal alteration can have a potential impact on the ability to distinguish human from nonhuman bone histologically if the structures are obscured because of corresponding color changes, or if the size of the osteons are altered due to temperature.

#### 1.1.2. Age-at-Death Estimation

In addition to human/nonhuman differentiation, histological analysis of cortical bone can help with the estimation of age-at-death. This histological estimate of age-at-death can be used in conjunction with other gross morphological age indicators to gain a more holistic age-at-death estimate [10,11] or, for highly fragmented remains, can be used as an alternative to gross age estimation [11,43]. Regardless of the method, adult histological age-at-death estimation from cortical bone is based generally on two principles: (1) osteons accumulate as age increases and (2) the amount of cortical bone available for osteon creation decreases with age. Although methods for estimation of age-at-death from histological analysis differ in terms of variable inclusion, statistical approach, and sampling area, most histological age estimation of bone is conducted using cross-sectional samples of undecalcified cortical bone from either the femur [12,13,14,15,16,17,18,19,20,21,22,23,24,25,26,27] or rib [11,28,29,30,31,32,33] and evaluating how much of the cross-section is populated with osteons either whole or fragmentary. 

In addition to histological age-at-death estimation from cortical bone, age-at-death can also be estimated from microscopic observation of tooth cementum annulations (TCA) [34,41]. This approach typically uses transverse cross-sections of a single-rooted tooth root to estimate age. TCA is based on the fact that after tooth eruption into the dental arcade, seasonal layers of cementum are laid down each year–a dark/opaque layer in the winter and a light/translucent layer in the spring [36,40,42]. To calculate the age-at-death from the tooth, analysts typically count each winter band visible in the cementum [35]. This number is then added to the average age of eruption for that particular tooth type, and an estimate of age-at-death is made [34,35,36,41]. Some TCA methods rely solely on polarized or bright light microscopy and digital imaging [34,35,36,38,40] while others have used SEM analysis [39,41,42]. 

Overall, each of these histological applications for applied casework are based on the successful observation and quantification of histological structures. However, without knowing how thermal alteration can affect histological structure in bones and teeth, these methods cannot be reliably applied to thermally altered remains in applied settings.

### 1.2. The Effect of Thermal Alteration on Skeletal Histology

The initial description of histological bone changes due to thermal alteration was described in 1941 by Forbes [44], in which he outlines the general obstruction of osteon lamellae over time with burning and the retained visibility of osteocyte lacunae post burning. Few papers since then have experimentally examined the relationship between burning temperature and/or time on the histological structures of bone [45,46,47,48,49,50,51,52,53], and none known to the authors that include actualistic whole-body scenarios. Some researchers have specifically tested histological aging methods on burned archaeological bone [48], however without known context of the burning event, the temperature, or the timing, it is difficult to come to definitive conclusions about applicability of aging methods on presumed burnt bone. 

Of the papers that have looked at histomorphometric changes in cortical bone due to thermal alteration, results vary as to the effect of burning on histomorphometric structures. Some papers indicate that the microscopic structures such as osteons expand through burning [45,54], while others claim structures contract [47,49,51]. The discrepancy in whether structures expand or contract could be related to the temperature of the fire and the mineral composition of the bone itself pre-burning such that the hydroxyapatite crystals in mineralized bone expand in size during initial burning, only to contract with increased burn time [52]. These previously published studies [45,47,49,51,54] however, did not examine an overlapping range of burning temperatures or burning times, meaning that there is currently no consensus as to why their results differ (see Carroll and Squires [53] for details on the inconsistencies of previous experiments and results). Apart from bone experiments, other researchers have also experimented on dental root microstructure to examine the effects of thermal alteration [35,55], and found higher temperatures impacted the visibility of cementum layers, inhibiting analysis for age estimation. All of these experiments on cortical bone and dental roots, however, used excised bone or extracted teeth which prevented the observation of the effects that surrounding soft tissues might have on the temperature of the bones and teeth and/or histological structures. 

Given the current gaps in the literature regarding histological thermal alteration experiments, this paper examines the effect of thermal alteration on bones and teeth through whole-body experimentation in order to evaluate the real-world applicability of histological age-at-death estimation methods on thermally altered human remains. 

## 2. Materials and Methods

### 2.1. Experimental Design

In order to investigate the effect of whole-body burning on the histological structures of human bones and teeth, six donors from the Forensic Anthropology Center at Texas State (FACTS) were placed in various fire-death scenarios at the Forensic Anthropology Research Facility (FARF) at Texas State in San Marcos, Texas. Each of the donors or donors’ next-of-kin consented to the possibility of being utilized for advanced or traumatic research processes prior to experimentation. The complete demographic description of each donor and their location of placement are listed in Table 1. All donors died of natural causes and none suffered from metabolic disease that contributed to their cause of death. Locations of donor placement included supine in a fresh state of decomposition laid over the folded back seats of a 2002 Chevrolet Trailblazer (*n* = 1), supine in a fresh state of decomposition on a pyre of pallets in a burial pit (*n* = 1), supine in a mummified state of decomposition on a pyre of pallets in a burial pit (*n* = 1), and inside temporary building structures referred to as pods (*n* = 3). Each of the experimental pods was outfitted to replicate a room in a residence. Within the pods, one donor was placed supine in a fresh state of decomposition on a couch, while two donors were placed supine in a fresh state of decomposition on their respective beds. The mummified donor in the pit reached a mummified state naturally, as she was placed in a fresh state of decomposition on the ground surface at FARF two months prior to being used for this study. 

**Figure 1:**
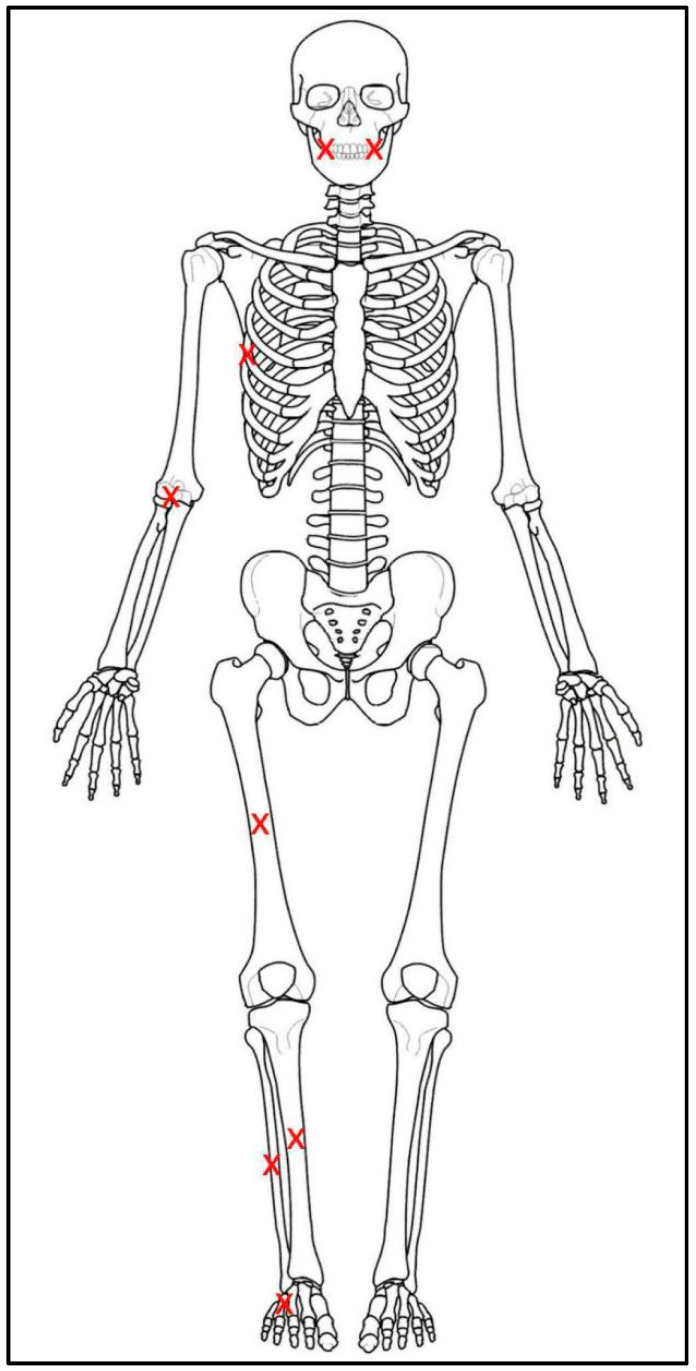
Approximate location of each thermocouple placement on the donors during burning displayed as a red Xs on a skeletal illustration. Each thermocouple was inserted into the flesh to be near the bone and wired into place. The thermocouples in the oral cavity were threaded up through the throat and held in place by the tissue within the mouth. The thermocouples on the ribs, thigh, and foot were used to estimate the temperature of the bone samples taken post burning. The thermocouples in the mouth were used to estimate the temperature of the tooth roots.

Once the donors were in position, probe thermocouples were inserted within the right side of the donors, one each within the elbow (antecubital fossa), rib cage (near the midshaft of the 6th rib), thigh (near the midshaft of the femur), shin (near the midshaft of the tibia), calf (also near the tibial midshaft), foot (near the 5th metatarsal), and two in the mouth (one laterally in the oral vestibule between the cheeks and gums, the other in the midline of the oral cavity) to capture temperature changes near the bone and teeth during burning (Figure 1). Thermocouples were also placed within the structures themselves to capture temperature fluctuation outside of the body during burning. The fires were set with the help and supervision of local fire departments utilizing fire starters within the individual structures and at the bottom of the pallet pyre in the case of the pit fire. All fires in the pods were extinguished with water through built-in sprinkler systems and all fires in the pit and the vehicles were extinguished with water applied via firehose to mimic actual forensic burn scenarios. Temperatures were captured via thermocouples continuously from the moment of ignition until the fire was extinguished. Total burn times for the pods were based on consultation with the fire departments on national response times for firefighters to mimic actual forensic scenarios, while burn times for the car and the pit were based on observation of body changes and available research time (Table 4). 

The degree of burning for each donor was assessed using the Glassman and Crow scale [56] which divides thermal alteration to human remains into five levels based on the degree of skeletal destruction (Table 2). This scoring was analyzed for each donor in order to allow for future researchers to correlate the survival of histological structures with the degree of thermal damage between experiments or real-world scenarios in situations where temperature cannot be recorded or determined.

#### 2.1.1. Bone Samples

The histological bone samples for this study were taken both prior to and after controlled burning and included approximately 1–2 cm lengths of bone from the midshaft of the femur, 6th rib, and an entire metatarsal. The samples were chosen to ensure there would be enough cortical bone to evaluate (1) general histomorphometric changes and (2) established age-at-death estimation methods. The samples taken before burning were removed from the donor’s left side with a hacksaw, shears, and/or scalpel. These samples were processed using standard warm water processing techniques and prepared for histological analysis using standard protocols [57]. These samples will be referred to throughout the paper as the “pre-burn sample”. The samples taken after burning were removed from the donor’s right side with a hacksaw, shears, and/or scalpel. These samples will be referred to throughout the paper as the “post-burn sample”. Table 3 summarizes all bone samples taken for each donor.

#### 2.1.2. Dental Samples

In addition to bone samples, post-burn dental samples were also removed from donors with preference given to single-rooted teeth. If available, canines were chosen first, followed by premolars, then incisors. Table 3 shows the exact dental samples taken after burning for each donor including tooth number as classified by the Fédération Dentaire Internationale (FDI) numbering system. Transverse cross-sections were taken from the middle 1/3 of each tooth root, ground to a thickness of 70–100 microns and mounted according to standard histological procedures [58].

### 2.2. Cortical Histomorphometric Analysis

In order to quantify the amount of thermal alteration present histologically, the cortical bone samples were imaged using a Leica DM6M light microscope with an automated stage at 100x magnification. The images were scaled, cropped, and exported into ImageJ [59] for analysis. To examine the effect of thermal alteration on histomorphometric measurements, the osteon and corresponding Haversian canal areas were measured throughout the cross-section of each sample. All cortices including the anterior, medial, posterior, and lateral cortices in the femur as well as the pleural and cutaneous cortices in the rib were measured to ensure coverage. There was no maximum osteon or Haversian canal count set for any element. Data was tested for normality using a Jarque-Bera test, extreme outliers were removed, and if necessary, the remaining data was transformed using a natural log (ln) transformation. Two-tailed t-tests were used to evaluate significant differences between pre and post-burn samples in the osteon area and Haversian canal area within each element. The alpha level was set at 0.05.

### 2.3. Cortical Age-at-Death Analysis

For a comparison of the effects of thermal damage on histological age-at-death estimation of bone, the Crowder and Dominguez method [25] was chosen to evaluate the femoral samples, while the Cho et al. method [29] was chosen to evaluate the rib samples. The Crowder and Dominguez method was chosen because of its statistical rigor and to evaluate its applicability to modern forensic samples, since it has not yet been validated. The specific variables for the femur method were collected using a combination of live-view and digital images from a Leica DM6M microscope with a counting reticle as detailed in Crowder’s [60] original National Institute of Justice report on which the Crowder and Dominguez method [25] is based. The final age-at-death estimates for the Crowder and Dominguez method were calculated using the keRley program [27,61], an online user interface that allows practitioners to apply Crowder and Dominguez’s histomorphometric variables for age-at-death estimation to a random forest modeling calculator.

All available samples were used in this analysis despite the degree of burning, which sometimes reduced the number of available variables for the Crowder and Dominguez method [25]. Due to the nature of the keRley interface, however, age-at-death estimates were still procurable provided at least one variable was available. The specific variables used for this method, including intact osteon population density 

(iOPD), fragmentary osteon population density (fOPD), anterior width of the femur (Ant.Wi), and average area of an osteon (On.Ar), were used for each sample’s age estimate. Unlike age estimation methods that utilize regression models, the Crowder and Dominguez method, when applied though keRley [27,61], uses random forest modeling with bootstrapping and training sets to minimize bias and minimize overfitting the data. The program then calculates a mean, median, and mode age from the aggregate of the trees to produce a 95% Interquantile Range (IR). The accuracy of the method on this sample was evaluated by assessing whether the known age-at-death fell within the predicted IR which is reported as a percentage of correct estimates over the total number of estimates in the results below (Table 8). 

The Cho et al. histological age-at-death estimation method [29], which utilizes linear regression models, was chosen for the rib because it is currently the most reliable well-established method available [11,57] that could provide a measure of whether thermal alteration affected the final age estimate. However, there was no prior expectation that it would perform well on either the pre- or post-burn sample given the known issues with the use of linear regression models for histological age estimation [11]. The specific variables for the rib method including osteon population density (OPD), average osteon area (On.Ar), and relative cortical area (rCt.Ar) were collected using a combination of live-view and digital images from a Leica DM6M microscope. All age estimates for the Cho et al. method were calculated using the Cho et al. unknown ancestry equation or fragmentary equation depending on the fragmentation of the available sample post-burning. Since the Cho et al. method is a linear regression model, the method produces a point age estimate which is comparable between samples. A root mean square error is provided from which an error range can be calculated, but previous research has shown that the accuracy of the Cho et al. method is limited by its linear regression approach [11], so accuracy was not tested in this experiment in the way it was for the femur. Alternatively, in order to focus on the effect of burning on the Cho et al. rib age estimate, the point age estimates for the rib were compared between the pre- and post-burn samples using *t*-tests (Table 9). To examine how well the Cho et al. method estimates age, the point age estimates for the pre- and post-burn samples were also compared to the donor known ages using *t*-tests (Table 9).

### 2.4. Dental Age-at-Death Analysis

Each dental slide was imaged using a Leica DM6M light microscope through a combination of live view and digital images. Since none of the teeth exhibit clear dental cementum layers through the entire depth of the cementum, the number of increments was extrapolated mathematically for age estimation following methodology proposed by Oliveira-Santos and colleagues [55]. This was done by measuring the thickness of a section of cementum (C) from the dentin/cementum border to the external border of the root. Within that section, the thickness of two pairs of discernable opaque and translucent lines (L) was also measured (Figure 5). The formula C/(L/2) was then used to estimate the number of years represented in a given section. That number was then added to the age of full eruption for that tooth according to AlQhatani and colleagues [62]. Age estimates were performed for each tooth and then averaged to yield a global average from the samples of each donor (Table 10). The utility of the method was evaluated by comparing the TCA global average estimate with the known age of the donor using *t*-tests (Table 10). In applied contexts there are issues with the applicability of the TCA method in forensic cases since there are no calculated statistical measure of error associated with this method, however the aim of this paper was to see if the available methods could be applied post-burning to support the development of statistically rigorous TCA methods in future research.

## 3. Results

### 3.1. Thermocouple Readings

The maximum burning temperature for the body thermocouples can be found in Table 4. These temperatures range from 25.8 °C to 1384.6 °C and are consistent with the burning temperatures from previous studies [53]. The hottest average temperatures were recorded within the mummified donor in the pit (D6) and the donor in the car (D1). The maximum burning temperatures for the structure thermocouples can also be found in Table 4. These temperatures range from 832 °C to 1115.71 °C. The absolute differences between the body thermocouples and the structure thermocouples range from 114.62 °C to 1044.53 °C. This highlights that the presence of soft tissue on remains has an effect on the burning temperature of bone, indicating histological investigations of thermally altered remains should use fleshed remains for studies meant to apply to fleshed bodies.

### 3.2. Cortical Bone Histomorphometric Analysis

**Figure 2:**
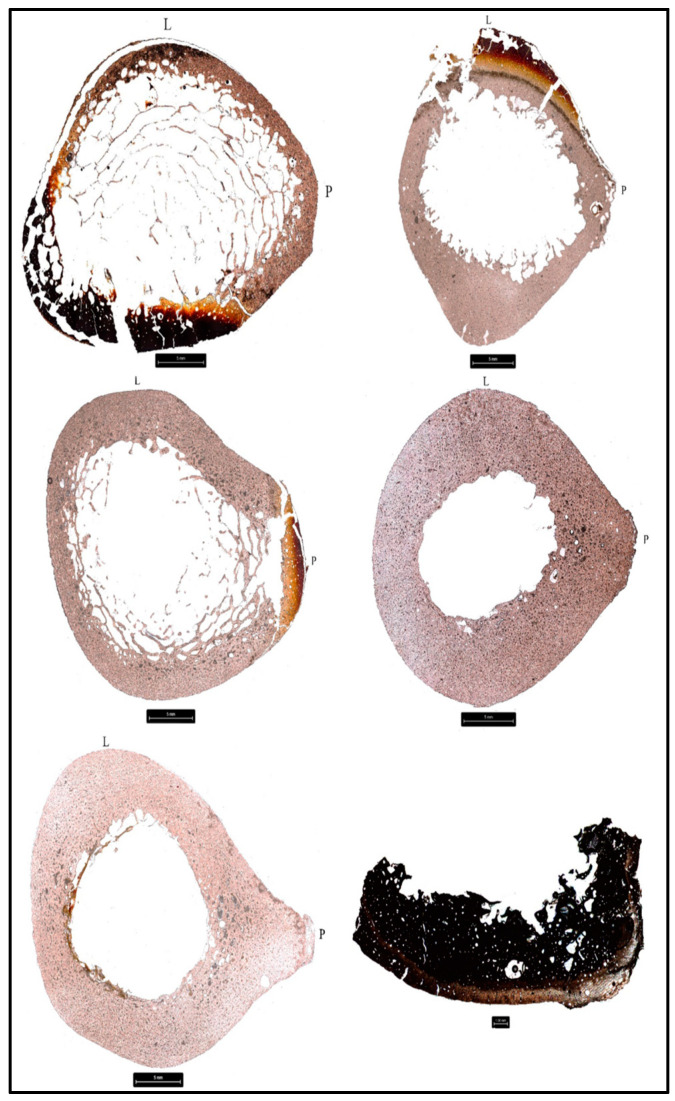
Histological sections taken at 50X from the femora of donors 1–6 post-burning with variation in burning patterns across the donors visible as yellow, red, black, and white discoloration. Top left: D1, top right: D2, middle left: D3, middle right: D4, bottom left: D5, bottom right: D6. L: lateral, P: posterior.

**Figure 3:**
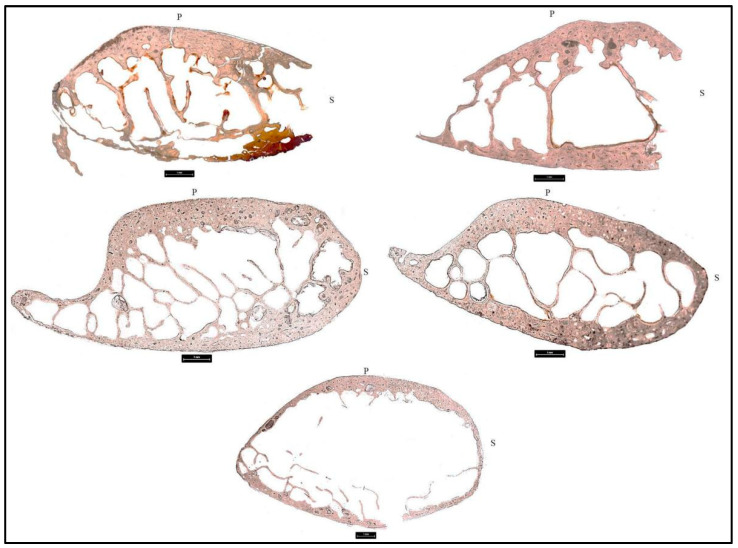
Histological sections taken at 100X from the ribs of donors 1–5 post-burning with burning visible as orange and red discoloration in D1. Top left: D1, top right: D2, middle left: D3, middle right: D4, bottom: D5. No rib was recoverable from D6. S: superior, P: pleural.

**Figure 4:**
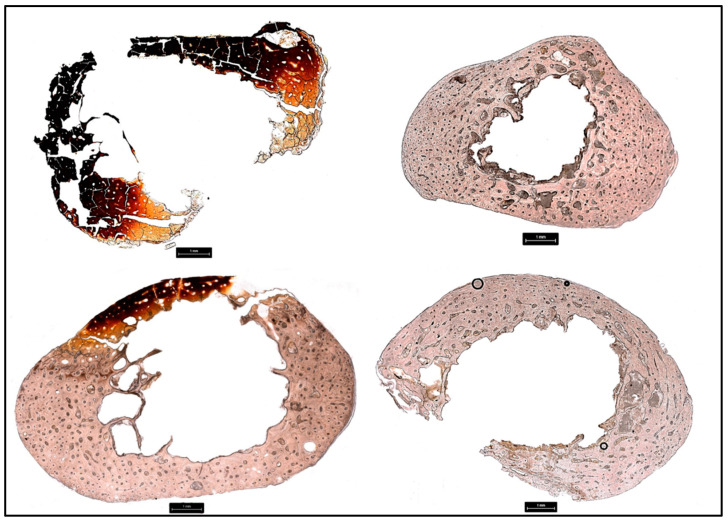
Histological sections taken at 100× from the metatarsals of Donors 2–5 post-burning with burning visible as yellow, orange, red, and black discoloration in D2 and D4. Top left: D2, top right: D3, bottom left: D4, bottom right: D5.

Each donor was scored according to Glassman and Crow [56] for the degree of burning (Table 5). The description of each excised bone sample before histological preparation was also evaluated for color changes and the results are available in the supplementary material (Table S1). Each sampled element of each donor was evaluated for histomorphometric changes including mean osteon area and mean Haversian canal area (Figure 2, Figure 3 and Figure 4, Table 6 and Table 7). Two-tailed t-tests were performed to evaluate significant histomorphometric differences between the pre- and post-burn samples for osteon area (Table 6) and Haversian canal area (Table 7). The results from these tests organized by donor are discussed below (Table 6 and Table 7). The total number of osteons and Haversian canals measured for each sample were limited by the preservation of the post-burn samples. 

#### 3.2.1. Donor 1 (D1)

The temperature of the structure reached a maximum of 1116 °C measured in the middle of the floorboard. The thermocouple in the thigh reached a maximum temperature of 1054 ℃. The thermocouple for the thorax nearest the rib reached 1273 °C. The thermocouple for the foot nearest the metatarsal reached 1385℃. The thermocouples within the body (thorax and foot) of the donor reached higher maximum temperatures than the thermocouples for the car itself. The entire donor post burning was mostly calcined in the appendages and cranium, but still exhibited some soft tissue in the trunk. No metatarsal was recoverable post-burning for analysis.

The femora showed significantly larger osteons (Table 6), but no significant difference in Haversian canals after burning (Table 7). The ribs showed significantly smaller osteons (Table 6) and Haversian canals post-burn (Table 7). The osteons and Haversian canals in the metatarsals could not be analyzed because no metatarsal data was recoverable post-burn. These results are not consistent with previous research in that each element exhibited different post-burn changes that did not appear to follow any pattern with thermocouple temperature. The thermocouples inserted into the thigh and near the sixth rib each reached over 1000 degrees Celsius and burned for the same amount of time (42 min), however the changes exhibited in the corresponding bone were inconsistent.

#### 3.2.2. Donor 2 (D2)

The temperature of the pit reached a maximum of 1037 °C measured along the bottom of the pit. The thermocouple in the thigh reached a maximum temperature of 718 °C. The thermocouple in the thorax nearest the rib reached a temperature of 955.7 °C. The thermocouple in the foot reached a temperature of 987.6 °C. The thermocouples in the middle and side of the mouth reached a temperature of 549.3 °C and 667.3 °C, respectively. The thermocouples within the body never reached temperatures higher than the pit itself. 

The femur showed no significant difference in osteon size between pre- and post-burn samples (Table 6), but Haversian canals were significantly larger in the post-burn sample (Table 7). The osteons and Haversian canals in the ribs showed no significant differences between the pre- and post-burn samples (Table 6 and Table 7). No osteons were measurable in the post-burn metatarsal data due to discoloration of the bone from burning, but the metatarsal exhibited significantly larger Haversian canals in the post-burn sample (Table 7). 

#### 3.2.3. Donor 3 (D3)

The maximum temperature of the structure was measured to be 881 °C within the pod. The thermocouple in the thigh reached a maximum temperature of 440 °C. The thermocouple in the thorax nearest the sixth rib reached a temperature of 324 °C. The thermocouple in the foot reached a temperature of 781 °C. The thermocouples in the middle and side of the mouth reached a temperature of 821.3 °C and 860.8 °C, respectively. The thermocouples in the lower leg of the body (in the shin and calf) reached higher temperatures than the thermocouple in the structure itself at 931 °C and 886 °C respectively. 

The osteons and Haversian canals in the femora and the ribs showed no significant difference between pre- and post-burn samples (Table 6 and Table 7). In the metatarsal, osteons were significantly larger in the post-burn sample than in the pre-burn sample (Table 6). There was no significant difference in Haversian canal size in the metatarsals between the pre- and post-burn samples (Table 7). 

#### 3.2.4. Donor 4 (D4)

The maximum temperature of the structure was measured to be 833 °C. The thermocouple in the thigh reached a maximum temperature of 30 °C. The thermocouple in the thorax nearest the 6th rib reached a temperature of 532 °C. The thermocouple in the foot reached a temperature of 827 °C. The thermocouples in the middle and side of the mouth reached a temperature of 479.2 °C and 457.6 °C, respectively. The thermocouples in the body never reached as high a temperature as the thermocouple within the structure itself. 

The osteons and Haversian canals in the ribs and femora showed no significant differences between pre- and post-burn samples (Table 6 and Table 7). The osteons in the metatarsals showed no significant difference between pre- and post-burn samples (Table 6); however, Haversian canals in the metatarsal post-burn sample were significantly smaller than in the pre-burn sample (Table 7). When compared to D3 who burned for the same amount of time with similar thermocouple maximum temperatures, it is interesting that this donor exhibited a significant decrease in Haversian canal area, while D3 did not (Table 7). 

#### 3.2.5. Donor 5 (D5)

The maximum temperature of the structure was measured to be 1070 °C. The thermocouple in the thigh reached a maximum temperature of 46 °C. The thermocouple in the thorax nearest the 6th rib reached a temperature of 26 °C. The thermocouple in the foot reached a temperature of 818 °C. The thermocouples in the middle and side of the mouth reached a temperature of 1034.8 °C and 1072 °C, respectively. The only thermocouple that reached a higher temperature than the structure thermocouple was the thermocouple in the side of the mouth. 

The osteons and Haversian canals in the femora and ribs showed no significant differences between pre- and post-burn samples (Table 6 and Table 7). The osteons in the metatarsals showed no significant differences between pre- and post-burn samples (Table 6). The Haversian canals in the metatarsals were significantly larger post-burning than pre-burning (Table 7). Interestingly, the donor had a metal rod in the left thigh that replaced the femur which was discovered upon sampling. 

#### 3.2.6. Donor 6 (D6)

The temperature of the pit near D6 reached a maximum of 1080 °C. The thermocouple in the thigh reached a maximum temperature of 1045 °C. The thermocouple in the thorax nearest the 6th rib reached a temperature of 1074 °C. The thermocouple in the foot reached a temperature of 1063 °C. The thermocouple in the foot reached a temperature of 818 °C. The thermocouples in the middle and side of the mouth reached a temperature of 935.6 °C and 924.5 °C, respectively. The thermocouples within the body never reached max temperatures equivalent to the temperature measured in the pit itself. No rib, metatarsal, or tooth sample was recoverable. The only element identifiable post-burn and available for sampling was a fragment from the posterior femur. 

Due to the degree of destruction of bone, post-burn analysis of D6 could only be conducted on the femur. The osteons and Haversian canals were significantly smaller in the post-burn sample than in the pre-burn sample (Table 6 and Table 7). Since the posterior femur was the only post-burn sample recoverable, it is possible the extreme osteon size difference is in part due to the size of the osteons in the posterior femur rather than changes based solely on thermal alteration. 

### 3.3. Cortical Age-at-Death Estimation

#### 3.3.1. Crowder and Dominguez [25] Femur Aging Method

The final age-at-death estimates for the Crowder and Dominguez method [25] are presented in Table 8, including which variables were used for the final estimate. The accuracy of the method was 100% for the preburn sample and 90% for the post-burn sample. The raw data for the Crowder and Dominguez method [25] is available in the supplementary material Table S2.

Of the two femora that exhibited significant size differences in osteon area pre- and post- burning, only one (D1) was usable for the Crowder and Dominguez method [25] due to the lack of anterior cortex in the other (D6). In order to test the utility of the method for thermally altered remains when On.Ar was potentially altered, the samples were rerun through the keRley [27] program without On.Ar. The results show that without On.Ar, the predicted age ranges post-burn still have 90% accuracy (Table 8). Notably, removing the On.Ar measurement from D1 did not improve the age estimation, which suggests that for this individual, intact osteon population density was out of the normal range. This shows that for thermally altered remains, the Crowder and Dominguez method is applicable and reliable even if On.Ar is immeasurable. 

#### 3.3.2. Cho et al. [29] Rib Aging Method

The final age-at-death estimates for the Cho et al. method [29] calculated using the unknown ancestry and fragmentary equations are presented in Table 9. The raw data for the Cho et al. method [29] is available in the supplementary material Table S3. No significant differences were found between pre- and post-burn estimates (t(4) = −1.94, *p* = 0.125). Significant differences were found between the estimated and known ages (t(10) = 6.88, *p* < 0.001) inclusive of pre- and post-burn samples.

Although there was a significant difference between the estimated and known ages, thermal alteration did not have a significant effect on the estimates. This indicates that although this particular method does not perform well on this sample, which was expected based on previous research into the utility of linear regression to estimate histological age at death [11], improved histological age-at-death estimation methods in the future that incorporate random forest modeling like the keRley [27,61] interface or other advanced statistical models could be applied to rib histomorphometry regardless of thermal alteration. It is also noteworthy that the post-burn rib samples in these experiments scoring up to a level 3 on the Glassman and Crow scale [56] were not significantly altered despite high temperatures recorded via thermocouples in both the structures and the thorax. It is possible that in contexts where ribs have undergone thermal alteration resulting in extensive damage such as cremation, histomorphometric variation might be greater between pre- and post-burn samples which would produce significantly different results.

#### 3.3.3. Dental Age Estimation

**Figure 5:**
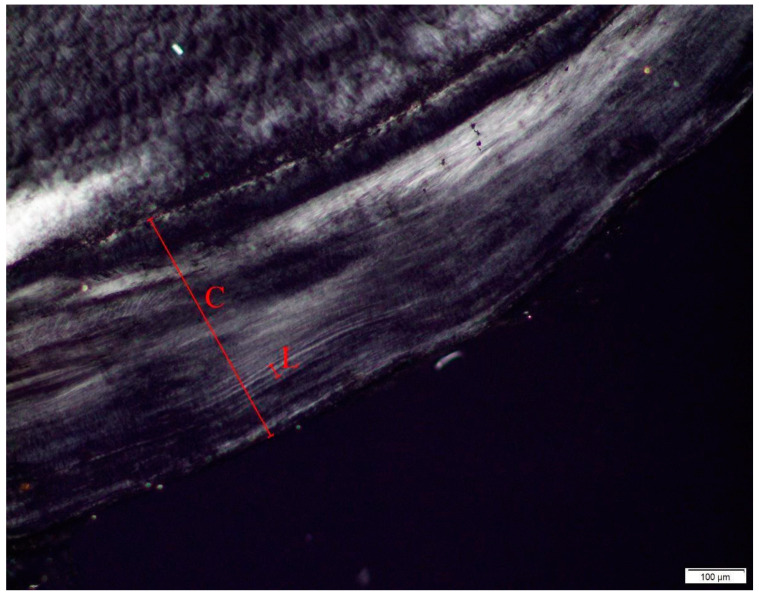
Histological section of a dental root from D2 illustrating how tooth cementum analysis (TCA) is applied. In the image, L is the length of two discernable annulations and C is the length of the thickest section of cementum. An estimate of the number of years represented as annulations in the tooth root is then calculated by dividing C by L/2.

Dental samples were recoverable from four of the donors (Table 3) and analyzed for TCA age estimation (Figure 5). Microscopic examination of the dental slides confirmed that the cementum had not undergone significant thermal alteration, displaying none of the hallmarks of burned cementum such as opacity, radial cracking, and an overall ‘gritty’ appearance [63]. Some areas of cementum were clear enough to identify increments/annulations, however, nowhere were increments clear enough through the entire thickness of cementum to warrant traditional counts for age estimation. The results of the TCA showed that the method underestimated known age in 75% of the donors with an average difference of −3.96 years between estimated and known age (Table 10). When compared using a t-test, the known ages were not significantly different from the post-burn estimated ages (t(3) = −0.74, *p*= 0.512). The results of this study show that cementum can still be analyzed for age estimation after undergoing thermal alteration, however, TCA age estimation for older individuals is complicated by the poor visibility of cementum lines. This difficulty in cementum visibility for older individuals is a known issue with TCA analysis for season-at-death estimation and future studies should be aware of the issues of using older individuals to test the TCA age estimation method [64].

## 4. Discussion

In these experiments, histomorphological changes in bone were inconsistent across scenarios and temperatures. Results show that histomorphometric changes were seen at the lowest recorded temperature of 781 °C in the metatarsal of D3. Not all recorded temperatures over 781 °C, however, resulted in histomorphometric changes, which differs from previous experimental research [53]. This is possibly related to the amount of tissue at each location, whereby higher temperatures are required to affect bone located deeper within soft tissue [1].

When histomorphometric changes did occur in the post-burn samples, the changes show no consistent pattern. In two individuals, osteon size increased in the post-burn femur (D1) and metatarsal (D3); and in two individuals, osteon size decreased in the post-burn rib (D1) and femur (D6). Decrease in Haversian canal size post-burn was observed in one rib (D1), one metatarsal (D4), and one femur (D6). Haversian canal increases were observed in one femur (D2) and two metatarsals (D2 and D5). These inconsistent results suggest that histomorphometric changes do occur during thermal alteration in skeletal remains, but the change is unpredictable using only temperature as a predictor of change, which corresponds with previous research reported by Carroll and Squires [53]. These results also show the amount of thermal variation present within different parts of the body when compared to structure temperature and indicate that soft tissue shielding can have a large effect on temperature of the bone, especially on histological preservation. 

**Figure 6:**
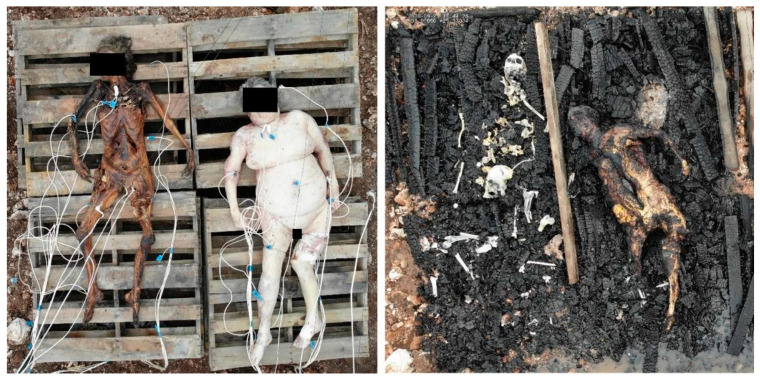
**Left**: Donors 6 and 2 on the same pyre, but in different stages of decomposition, pre-burning. **Right**: Donors 6 and 2 on the same pyre, post-burning. In the post-burn image, the resultant thermal destruction is more pronounced for D6 who was mummified pre-burn (**left**), than for D2 who was in a fresh state of decomposition pre-burn (**left**). Differences in preservation were also seen in the histological preservation post-burning (Figure 2, Figure 3 and Figure 4).

These results suggest it is likely that the use of whole, fleshed remains affected the lack of histological changes seen in these experiments when compared to previous studies which relied on excised bone [45,46,47,48,49,50,51,52,53]. This is possibly explained by the hypothesis that the state of the bone pre-burning affects the amount of thermal destruction it undergoes during burning [65]. An excellent example of this differential burning due to tissue state can be seen in the burn scenario between D2 and D6 where each donor was burned on the same pyre (Figure 6), but one was in a state of fresh decay (D2) and one was already mummified (D6). The resultant thermal destruction to the body for each donor was noticeably different in that the donor in a fresh state (D2) classified as Level 3, major destruction on the Glassman and Crow scale [56], while the donor in a mummified state (D6) classified as Level 5, cremation. In addition to tissue shielding causing differential thermal destruction, it is also possible the loss of moisture content in the mummified remains [66] contributed to the differential burning. It is also possible that the position of the donors (supine) may also affect the pattern of burning, and individuals who are seated or laying in a prone position may exhibit variation in burning pattern. 

The age estimation methods tested in this study performed similarly between pre- and post-burn samples which supports the use of histological age-at-death estimation for thermally altered remains. Specifically, this study showed that for individuals scoring up to Level 3 on the Glassman and Crow scale [56] histological samples from the femur, rib, metatarsal, and tooth roots were still recoverable from fire scenes and able to be analyzed. Additionally, even D6, which scored a Level 5 on the Glassman and Crow scale, yielded identifiable fragments with visible histomorphology. Although not explicitly tested in these experiments, similar to Cattaneo et al. [49], this histomorphological preservation at Level 5 suggests that histomorphology-based analyses such as human vs. nonhuman differentiation, season-of-death, and histotaphonomic investigations can be conducted on thermally altered remains, although histomorphometric analyses may be skewed. 

Specifically, this study showed that the Crowder and Dominguez method [25] when applied through the keRley program [27,61] accurately predicts age-at-death when anterior width of the femur is included as a variable. Although the sample size was small, these results support future testing of this method in applied contexts and suggests that a random forest approach to histological age-at-death estimation can improve upon traditional linear regression models. These results also directly support the development of random forest modeling for histomorphometric age-at-death estimation of the rib.

Although the TCA global average estimates were not statistically significantly different from the known ages, this TCA method is hard to use in applied contexts where a measure of statistical error is required, such as in a forensic case. Similar to the results from the ribs using the Cho et al. method [29], these dental results suggest that future TCA methods which employ more rigorous statistical modeling with some measure of reportable error could be used effectively on thermally altered remains. 

One potential issue in the experimental design of this study is the extant symmetry between the histomorphometry of the left and right elements from within a donor. Previous research has shown no significant differences in histomorphometry between the left and right sides of ribs [67] or femora [16], but no established research has been done on metatarsals. However, research into the heritability of osteon area in some mammal bones [68] suggests osteon area is genetically constrained, which further supports the assumption of bilateral symmetry in this study. Additional limitations of this study include the small sample size (N = 6) and the uncontrolled conditions relating to the size, temperature, intensity of the fire, as well as the various body positioning. This inconsistency was unavoidable due to the nature of the fire death scenarios and could be a factor in comparing these results to future experimental fire studies. The use of the Glassman and Crow scale was an attempt to allow future researchers to compare histological results according to the degree of burning of the remains found in context, rather than comparing exact fire burning scenarios.

## 5. Conclusions

This was the first study to use whole-body human donors in place of excised bone to examine the effect of thermal alteration on bone histomorphometry and tooth root annulation visibility. The results of this study show that thermal alteration had an unpredictable effect on histomorphometry of bone in regard to temperature of the fire, element examined, and time of burning used in this study. The Crowder and Dominguez femur age-at-death estimation method [25] when applied via the keRley program [27,61] performed well on both the pre- and post-burn samples. The point age estimates for the Cho et al. rib age-at-death estimation method [29] did not differ significantly pre- and post-burning. The TCA results showed no significant differences between the estimated and known ages, which supports the use of TCA for age-at-death estimation in burned human remains. Every donor, regardless of degree of burning, was able to produce at least one element for histological bone analysis. The ability to discern histomorphological structures in even severely burnt bone supports the use of histological analysis of thermally altered bones and teeth in applied contexts for human vs. nonhuman differentiation, age-at-death estimation, and histotaphonomic interpretation. 

Future research will attempt to expand sample sizes to see if any discernible pattern can be deduced between burning temperature, time, body position, and histomorphometric changes. Future research will also examine the effects of thermal alteration on histotaphonomic signatures.

## Figures and Tables

**Table 1:** Demographics of donors and donor placement location.

Donor Number and Description	Sex	Age	Fire-Death Scenario
D1 Car Donor	F	84	Car
D2 Fresh Pit Donor	F	81	Pit, Fresh
D3 Pod Donor 1	F	74	Pod 1, Couch
D4 Pod Donor 2	F	61	Pod 2, Bed
D5 Pod Donor 3	M	65	Pod 3, Bed
D6 Mummified Pit Donor	F	67	Pit, Mummified

**Table 2:** Glassman and Crow Scale (1996) levels and descriptions.

Level	Stage	Description
1	Recognizable	Typically smoke death
2	Possibly recognizable	Charring on elements such as hand/feet, genitalia
3	Non-recognizable	Major destruction of head and extremities
4	Extensive burn destruction	Skull and extremities are severely fragmented or missing
5	Cremation	Little or no tissue remains

**Table 3:** Description of pre- and post-burn samples listed by donor. Tooth numbers correspond to the FDI nomenclature.

Donor	Pre-Burn Bone Samples	Pre-burn tooth samples	Post-Burn Bone Samples	Post-Burn Tooth Samples
D1	Femur, Rib, Metatarsal	N/A	Femur, Rib	N/A
D2	Femur, Rib, Metatarsal	N/A	Femur, Rib, Metatarsal	14, 11, 43
D3	Femur, Rib, Metatarsal	N/A	Femur, Rib, Metatarsal	23, 32
D4	Femur, Rib, Metatarsal	N/A	Femur, Rib, Metatarsal	21, 23, 25
D5	Femur, Rib, Metatarsal	N/A	Femur, Rib, Metatarsal	43, 44, 45
D6	Femur, Rib, Metatarsal	N/A	Femur	N/A

**Table 4:** Maximum burning temperature in degrees Celsius recorded for each thermocouple in the body and in the fire-death scenario listed by donor as well as the total duration of burning time in minutes and the mean temperature in degrees Celsius for the entire body during burning.

Donor	Mouth (Middle)	Mouth (Side)	6th Rib	Elbow	Thigh	Shin	Calf	Foot	Structure	Burn Duration (min)	Mean Burn Temp
D1	801.8	795.2	1273.0	880.5	1053.8	1390.4	959.3	1384.6	1115.7	42	487.7
D2	549.3	667.3	955.7	923.8	718.3	823.0	314.5	987.6	1036.6	53	206.6
D3	821.3	860.8	324.4	307.6	440.4	931.4	885.6	781.0	880.5	32	193.0
D4	479.2	457.6	532.0	83.5	29.8	105.8	731.2	827.2	832.8	32	152.0
D5	1034.8	1072.0	25.8	452.4	45.9	756.0	684.4	817.5	1070.4	21	251.2
D6	935.6	924.5	1074.1	1014.0	1045.3	1020.8	1014.6	1063.2	1080.2	53	352.9

**Table 5:** Glassman and Crow (1996) body scores for each donor after burning.

Donor	Body Score
D1	4
D2	3
D3	2
D4	2
D5	2
D6	5

**Table 6:** Mean osteon area for pre- and post-burn samples as well as the paired sample t-test results showing the comparisons of mean osteon area (On.Ar) from pre- and post-burn samples by donor.

Donor	Element	Number of Osteons Measured	Pre-Burn		Post-Burn		On.Ar *t* Statistic (df)
			Mean	Standard Deviation	Mean	Standard Deviation	
D1	Femur Rib Metatarsal	19 39 0	0.042 0.027 N/A	0.017 0.010 N/A	0.055 0.020 N/A	0.019 0.009 N/A	−2.7 (18) * 2.31 (39) * N/A
D2	Femur Rib Metatarsal	29 21 0	0.035 0.033 N/A	0.010 0.014 N/A	0.040 0.034 N/A	0.010 0.013 N/A	−1.8 (28) −0.22 (20) N/A
D3	Femur Rib Metatarsal	40 49 39	0.030 0.025 0.020	0.010 0.010 0.010	0.030 0.025 0.030	0.010 0.012 0.010	−0.23 (39) 0.04 (48) −3.46 (38) **
D4	Femur Rib Metatarsal	40 41 41	0.045 0.030 0.035	0.014 0.020 0.011	0.049 0.030 0.033	0.021 0.010 0.011	−0.71 (39) −0.73 (40) 0.83 (40)
D5	Femur Rib Metatarsal	40 42 39	0.050 0.030 0.038	0.020 0.010 0.020	0.050 0.030 0.030	0.020 0.010 0.010	−0.14 (39) −0.53 (41) 1.69 (38)
D6	Femur Rib Metatarsal	21 0 0	0.056 N/A N/A	0.020 N/A N/A	0.036 N/A N/A	0.014 N/A N/A	3.29 (20) ** N/A N/A

* indicates *p* < 0.05. ** indicates *p* < 0.01.

**Table 7:** Mean Haversian canal area for pre- and post-burn samples as well as paired sample t-test results showing the comparison of pre- and post-burn Haversian canal area (H.Ar) by donor.

Donor	Element	Number of Haversian Canals Measured	Pre-Burn		Post-Burn		H.Ar *t* Statistic (df)
			Mean	Standard Deviation	Mean	Standard Deviation	
D1	Femur Rib Metatarsal	20 40 0	0.0035 0.0020 N/A	0.0172 0.0013 N/A	0.0037 0.0012 N/A	0.0147 0.0010 N/A	−0.2 (19) 2.57 (39) * N/A
D2	Femur Rib Metatarsal	40 21 40	0.0338 0.0022 0.0017	0.0121 0.0014 0.0010	0.0380 0.0016 0.0024	0.0125 0.0011 0.0012	3.4 (39) ** 1.43 (20) −2.64 (39) *
D3	Femur Rib Metatarsal	40 49 41	0.0026 0.0020 0.0026	0.0018 0.0014 0.0017	0.0030 0.0017 0.0030	0.0024 0.0012 0.0020	−1.06 (39) 1.35 (48) −1.54 (40)
D4	Femur Rib Metatarsal	40 42 40	0.0038 0.0013 0.0048	0.0018 0.0007 0.0028	0.0036 0.0016 0.0037	0.0022 0.0009 0.0020	0.54 (39) −1.47 (41) 2.09 (39) *
D5	Femur Rib Metatarsal	38 42 38	0.0043 0.0020 0.0034	0.0031 0.0017 0.0022	0.0037 0.0018 0.0059	0.0021 0.0012 0.0030	0.92 (37) 0.75 (41) 4.94 (39) **
D6	Femur Rib Metatarsal	39 0 0	0.0033 N/A N/A	0.0020 N/A N/A	0.0019 N/A N/A	0.0010 N/A N/A	4.82 (38) ** N/A N/A

* indicates *p* < 0.05. ** indicates *p* < 0.01

**Table 8:** The results of the Crowder and Dominguez [25] femur age estimation using keRley [59] for the anterior femur. The results for pre- and post-burn samples are listed by donor showing the lower and upper limits of the 95% Interquantile Range (IR) as well as the mean estimate and the variables used. (iOPD = intact ostoen population density, fOPD = fragmentary osteon population density, On. Ar = Osteon Area, Ant.Wi = Anterior Width).

Donor	Known Age (Years)	Pre-burn Sample			Post-burn Sample			Post-Burn Sample Retest with no On.Ar		
		IR	Mean Estimate	Variables Used	IR	Mean Estimate	Variables Used	IR	Mean Estimate	Variables Used
D1	85	59–91 *	76	iOPD, fOPD,On.Ar, Ant.Wi	22–60	30	iOPD, On.Ar	24–50	29	iOPD
D2	81	61–93 *	78	iOPD, fOPD,On.Ar, Ant.Wi	57–86 *	68	iOPD, Ant.Wi	57–86 *	68	iOPD, Ant.Wi
D3	74	53–90 *	71	iOPD, fOPD,On.Ar, Ant.Wi	69–92 *	78	iOPD, On.Ar, Ant.Wi	69–85 *	77	iOPD, Ant.Wi
D4	61	50–92 *	73	iOPD, fOPD,On.Ar, Ant.Wi	55–82 *	67	iOPD, On.Ar, Ant.Wi	54–80 *	68	iOPD, Ant.Wi
D5	65	23–74 *	57	iOPD, fOPD,On.Ar, Ant.Wi	65–88 *	77	iOPD, Ant.Wi	65–88 *	77	iOPD, Ant.Wi
D6	67	56–85 *	71	iOPD, fOPD,On.Ar, Ant.Wi	No anterior femur recovered			No anterior femur recovered		

* indicates the known age fell within the predicted IR.

**Table 9:** Rib age estimates using Cho et al. [29] unknown ancestry equations showing the known age, osteon population density (OPD), and point age estimates for both the pre and post-burn samples by donor.

Donor	Known Age (years)	Pre-Burn		Post-Burn	
		OPD	Point Age (Years)	OPD	Point Age (Years)
D1	85	22.06	53.23	24.99	63.06 *
D2	81	21.69	48.92	22.4	51.83
D3	74	26.05	57.39	26.05	59.89
D4	61	21.47	46.16	22.5	49.36
D5	65	21.06	52.47	20.46	51.28
D6	67	23.63	61.58	N/A	N/A

* indicates point estimate was calculated from the Cho et al. [29] fragmentary equation due to taphonomic alteration.

**Table 10:** The data for the Tooth Cementum Annulation (TCA) analysis with final global age estimates as well as known age presented in the table. The number of tooth cementum annulations (TCA lines) calculated per tooth as well as the average age of tooth eruption for each tooth are also shown. Tooth numbers correspond to the FDI notation.

Donor	Tooth	Average Number of TCA Lines	Average Age of Tooth Eruption (Years)	Estimated Age Based on Tooth (Years)	Global Age Estimate (Years)	Known Age (Years)
D2	11	82.54	7.5	90.04	79.21	81
	43	56.89	11.5	68.38		
D3	23	39.14	12.5	51.64	56.31	74
	32	49.48	11.5	60.98		
D4	21	55.09	7.5	62.59	69.19	61
	23	63.29	12.5	75.79		
D5	43	52.58	11.5	64.08	60.42	65
	44	65.90	11.5	77.40		
	45	27.38	12.5	39.88		

## Data Availability

The thermocouple and additional histomorphometric data that supports these findings are available from the corresponding author upon reasonable request. Histomorphometric data used in the Crowder and Dominguez [25] and Cho et al. [29] methods are available in the Supplementary Material.

## Supplementary Materials

The following supporting information can be downloaded at https://www.mdpi.com/article/10.3390/biology11111569/s1, Table S1. A description of visible color changes on the excised bone and tooth samples and a description of visible color changes seen on the histological slide as visualized in Figure 2, Figure 3 and Figure 4. The descriptions are organized by sample and grouped by donor; Table S2. Raw data for the Crowder and Dominguez [25] Femur Ageing Method using the keRley [27] interface for pooled sexes with and without On.Ar; Table S3. Raw data for the Cho et al. [29] rib ageing method using the unknown ancestry equation adjusted to 50/50 ancestry groups.
