# Supplementary material for: The Effects of Experimental Whole-Body Burning on Histological Age-at-Death Estimation from Human Cortical Bone and Dental Cementum
Source: Biology (Basel). 2022 Oct 26;11(11):1569. doi: 10.3390/biology11111569 (PMC9687164; doi:10.3390/biology11111569)
Supplement: Supplementary file 1 [file biology-11-01569-s001.zip › biology-1915129-supplementary.pdf]

Supplemental Data

Table S1. A description of visible color changes on the excised bone and tooth samples and a description of visible color changes seen on the histological slide as visualized in Figures 2-4. The descriptions are organized by sample and grouped by donor.

Donor	Sample	Description of Excised Samples	Description of Histological Slides
D1	Femur	Primarily charred black with some browning	Black charring across anterior, medial, and lateral surfaces
	Rib	Some black charring on superior-cutaneous surface	Some orange discoloration on cutaneous surface
	Metatarsal	Not recovered	Not recovered
	Tooth	Not recovered	Not recovered
D2	Femur	Partial black charring	Some black and orange discoloration on lateral surface
	Rib	No change	No change
	Metatarsal	Primarily charred black	Mostly black discoloration with some orange
	Tooth 14	Partial black discoloration	No change
	Tooth 11 and 43	Partial white discoloration	No change
D3	Femur	Some black charring on posterior surface	Orange discoloration on posterior surface
	Rib	No change	No change
	Metatarsal	No change	No change
	Tooth 23 and 32	No change	No change
D4	Femur	No change	No change
	Rib	No change	No change
	Metatarsal	Some black charring with some brown discoloration	Some orange discoloration with some black charring
	Tooth 21	Partial white discoloration	No change
	Tooth 23 and 25	No change	No change
D5	Femur	No change	Some orange discoloration on endosteal surface
	Rib	No change	No change
	Metatarsal	Some browning	No change
	Tooth 43, 44, and 45	No change	No change
D6	Femur	Mostly calcined with some black charring	Primarily black discoloration across sample with some calcination on posterior surface
	Rib	Not recovered	Not recovered
	Metatarsal	Not recovered	Not recovered
	Tooth	Not recovered	Not recovered

Table S2. Raw data for the Crowder and Dominguez [25] Femur Ageing Method using the keRley [27] interface for pooled sexes with and without On.Ar.

Donor	Burn Status	OPD(I)	OPD(F)	Ant.Wi	On.Ar	95% IR	Mode	Mean	Median
D1	Pre	13.99818	16.45732	3.236525	0.043102911	59-91	91	76	77
D2	Pre	24.03846	135.5536	2.063088	0.039757313	61-93	61	78	81
D3	Pre	23.51279	17.83729	4.9178	0.033465286	53-90	61	71	69
D4	Pre	18.70819	15.67726	5.179197	0.038222012	50-92	85	73	74
D5	Pre	15.7872	4.865917	4.674802	0.047503163	23-74	56	57	56
D6	Pre	18.68607	12.29347	4.086559	0.043395625	56-85	63	71	71
D1	Post	10.11221	-	-	0.0535539	22-60	25	30	28
D2	Post	23.48726	-	2.66	-	57-86	61	68	63
D3	Post	16.93972	-	2.321325	0.031926646	69-92	74	78	77
D4	Post	18.85593	-	5.011415	0.03682746	55-82	65	67	66
D5	Post	14.82664	-	3.752907	-	65-88	78	77	78
D6	Post	NA	NA	NA	NA	NA	NA	NA	NA
Crowder & Dominguez [25] data and results for pooled sexes through keRley [27] program without On.Ar									
Donor	Burn Status	OPD(I)	OPD(F)	Ant.Wi	On.Ar	95% IR	Mode	Mean	Median
D1	Post	10.11221	-	-	-	24-50	25	29	26
D2	Post	23.48726	-	2.66	-	57-86	61	68	63
D3	Post	16.93972	-	2.321325	-	69-85	74	77	76
D4	Post	18.85593	-	5.011415	-	54-80	73	68	69
D5	Post	14.82664	-	3.752907	-	65-88	78	77	78
D6	Post	NA	NA	NA	NA	NA	NA	NA	NA

Table S3. Raw data for the Cho et al. [29] rib ageing method using the unknown ancestry equation adjusted to 50/50 ancestry groups.

Donor	Burn Status	OPD	Relative Ct.Ar	On.Ar	Point Age Estimate Unknown (2002)
D1	Pre	22.06	0.22	0.03	53.23
D2	Pre	21.69	0.39	0.03	48.92
D3	Pre	26.05	0.41	0.03	57.39
D4	Pre	21.47	0.50	0.03	46.16
D5	Pre	21.06	0.18	0.03	52.47
D6	Pre	23.63	0.28	0.04	61.58
D1	Post	24.99	NA	0.02	63.06*
D2	Post	22.40	0.31	0.03	51.83
D3	Post	26.05	0.36	0.02	59.89
D4	Post	22.50	0.43	0.03	49.36
D5	Post	20.46	0.18	0.03	51.28
D6	Post	NA	NA	NA	NA

*indicates the fragmentary equation was used
